# Supplementary material for: Spatial heterogeneity of physicochemical properties explains differences in microbial composition in arid soils from Cuatro Cienegas, Mexico
Source: PeerJ. 2016 Sep 8;4:e2459. doi: 10.7717/peerj.2459 (PMC5018672; doi:10.7717/peerj.2459)

Figure S1. Rarefaction curves showing 95% confidence interval of 16S rDNA TRFLPs for the four studied quadrants (A-D), displaying number of OTU detected versus number of samples analyzed per quadrant.

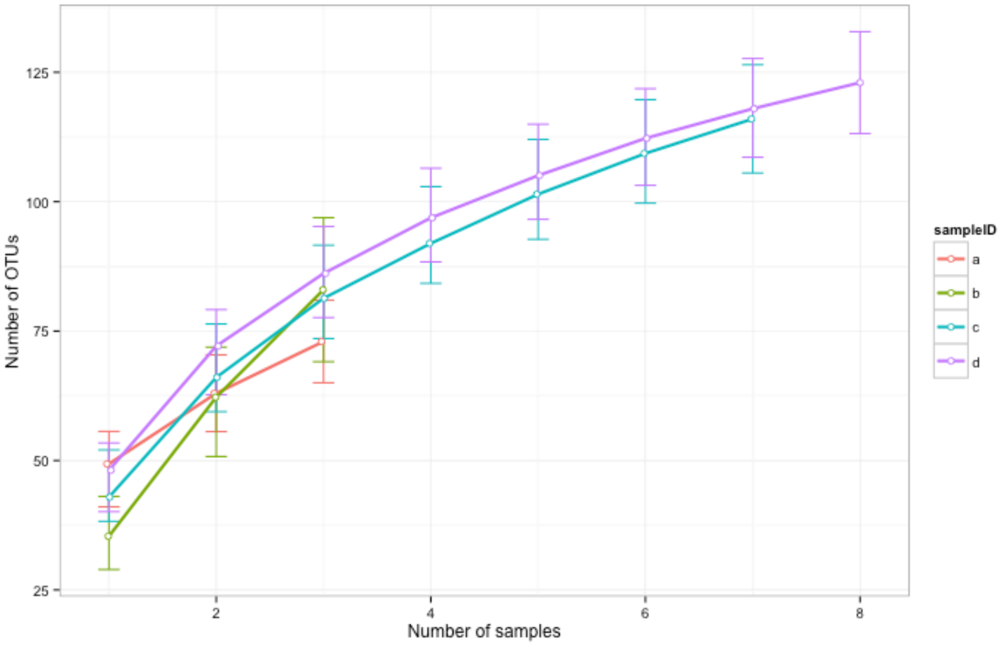

Supplement: Figure S1 — Showing 95% confidence interval of 16S rDNA T-RFLPs for the four studied quadrants (A-D), displaying number of OTU detected versus number of samples analyzed per quadrant [file peerj-04-2459-s002.pdf]
