# Supplementary material for: Spatial heterogeneity of physicochemical properties explains differences in microbial composition in arid soils from Cuatro Cienegas, Mexico
Source: PeerJ. 2016 Sep 8;4:e2459. doi: 10.7717/peerj.2459 (PMC5018672; doi:10.7717/peerj.2459)

Figure S2. Heatmap showing the similar microbial communities abundance from the TRFLPs profiles of the four quadrants (A: 3 samples; B: 3 samples; C: 7 samples; D: 8 samples). Bray-Curtis dissimilarity distances and the Ward's hierarchical clustering algorithm were applied in the analysis. OTUs with less than 1% of their maximum relative abundance were removed from the analysis (121 OTUs were removed from a total of 184 OTUs).

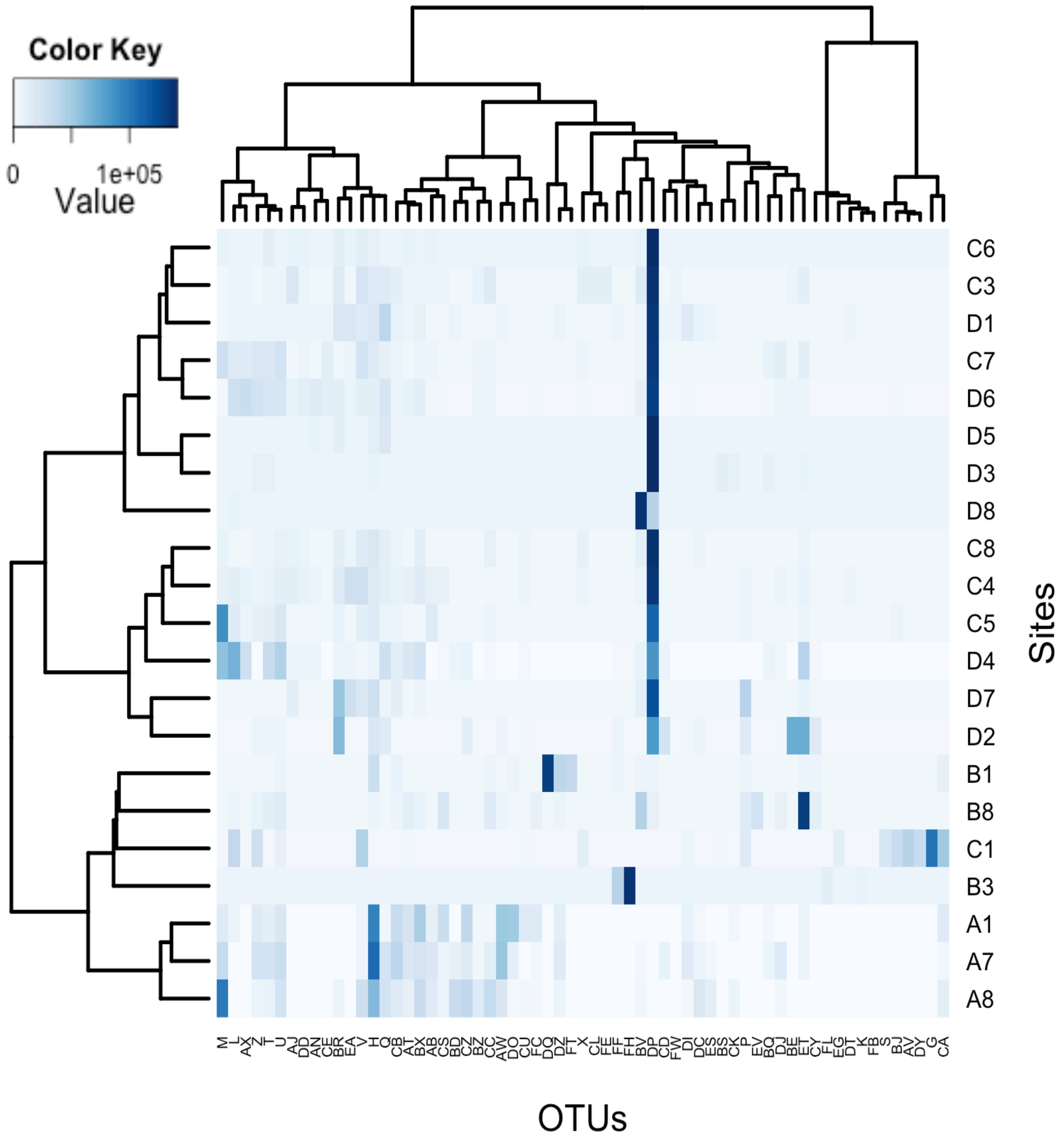

Supplement: Figure S2 — Showing the similar microbial communities abundance from the T-RFLPs profiles of the four quadrants (A: 3 samples; B: 3 samples; C: 7 samples; D: 8 samples). Bray–Curtis dissimilarity distances and the Ward’s hierarchical clustering algorithm were applied in the analysis. OTUs with less than 1% of their maximum relative abundance were removed from the analysis (121 OTUs were removed from a total of 184 OTUs). [file peerj-04-2459-s003.pdf]
