# Supplementary material for: Spatial heterogeneity of physicochemical properties explains differences in microbial composition in arid soils from Cuatro Cienegas, Mexico
Source: PeerJ. 2016 Sep 8;4:e2459. doi: 10.7717/peerj.2459 (PMC5018672; doi:10.7717/peerj.2459)

Figure S3. Cluster dendrogram of similar microbial communities from the TRFLPs profiles of the four studied quadrats (*A*: 3 samples; *B*: 3 samples; *C*: 7 samples; *D*: 8 samples) using Bray-Curtis dissimilarity distances and the Ward's hierarchical clustering algorithm.

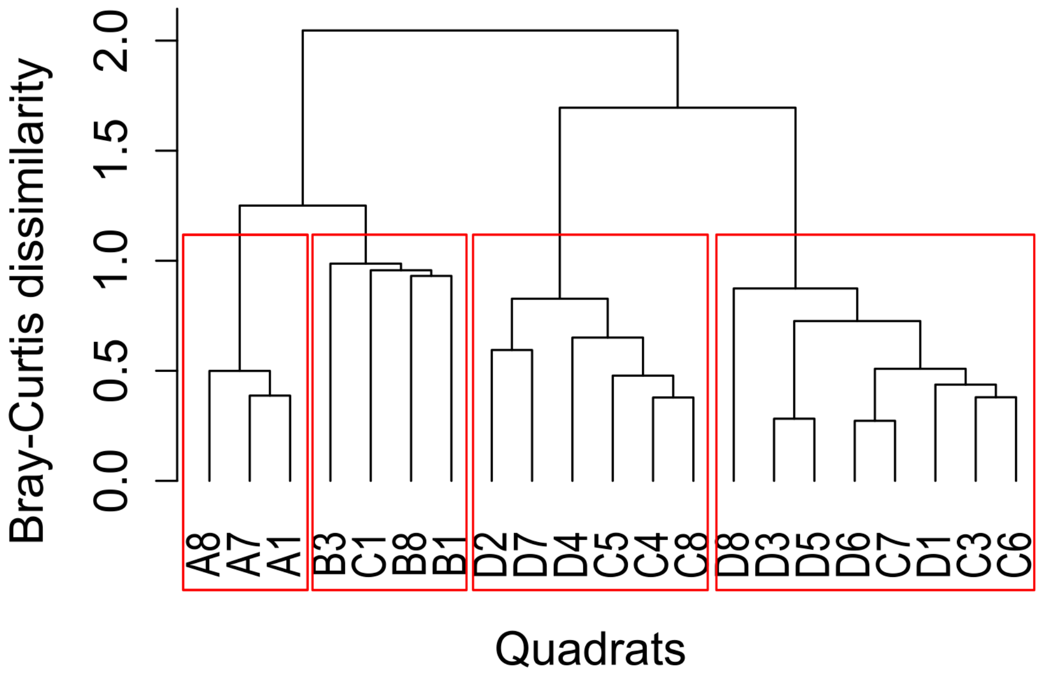

Supplement: Figure S3 — Cluster dendrogram of similar microbial communities from the T-RFLPs profiles of the four studied quadrats (A: 3 samples; B: 3 samples; C: 7 samples; D: 8 samples) using Bray-Curtis dissimilarity distances and the Ward’s hierarchical clustering algorithm. [file peerj-04-2459-s004.pdf]
