# Supplementary material for: Spatial heterogeneity of physicochemical properties explains differences in microbial composition in arid soils from Cuatro Cienegas, Mexico
Source: PeerJ. 2016 Sep 8;4:e2459. doi: 10.7717/peerj.2459 (PMC5018672; doi:10.7717/peerj.2459)
Supplement: Table S1 [file peerj-04-2459-s005.pdf]

SUPPLEMENTARY MATERIAL

Table S1. Pearson correlation coefficients among different soil parameters from the four quadrants within the Churince system in the Cuatro Cienegas Basin (Mexico).

	**TC**	**TN**	**C:N**	**TP**	**NH_4^+**	**NO_3^-**	**DOC**	**DON**	**DOC:NOD**	**DOP**	**pH**	**CE**	**Mg^{2+}**	**Ca^{2+}**	**Na^+**	**K^+**	**HCO_3^-**	**Cl^-**	**SO_4^{2-}**
**TC**	1																		
**TN**	0.194	1																	
**C:N**	0.335	-0.788**	1																
**TP**	0.178	-0.001	0.071	1															
**NH_4^+**	-0.201	-0.513**	0.421*	-0.062	1														
**NO_3^-**	0.050	-0.494**	0.479**	0.197	0.414*	1													
**DOC**	0.299	0.112	-0.049	0.278	-0.073	-0.045	1												
**DON**	0.073	0.460*	-0.278	-0.221	-0.251	-0.324	-0.123	1											
**DOC:NOD**	0.067	-0.232	0.104	0.244	0.266	0.130	0.616**	-0.734**	1										
**DOP**	0.052	0.031	-0.030	-0.582**	0.036	-0.365	-0.075	0.221	-0.032	1									
**pH**	0.009	0.190	-0.167	0.504**	-0.134	0.054	0.310	0.161	-0.015	-0.205	1								
**CE**	0.011	-0.118	0.097	-0.193	0.189	0.038	0.155	0.084	-0.071	0.244	0.241	1							
**Mg^{2+}**	0.192	0.137	-0.021	0.383*	-0.007	0.001	0.428*	-0.105	0.225	-0.066	0.554**	0.524**	1						
**Ca^{2+}**	0.410*	0.278	-0.074	0.432*	-0.340	-0.061	0.161	0.392*	-0.288	-0.313	0.202	0.000	0.198	1					
**Na^+**	0.009	-0.002	-0.071	0.533**	0.121	0.055	0.345	0.030	0.090	-0.368	0.567**	0.358	0.606**	0.367	1				
**K^+**	0.282	0.012	0.146	0.386*	0.157	0.265	0.259	0.063	0.000	-0.190	0.330	0.276	0.652**	0.488*	0.510**	1			
**HCO_3^-**	-0.211	-0.398*	0.348	-0.324	0.385*	0.063	-0.094	-0.300	0.200	0.224	-0.293	-0.083	-0.430*	-0.558**	-0.290	-0.388*	1		
**Cl^-**	-0.243	-0.322	0.267	-0.373	0.305	-0.048	-0.109	-0.225	0.174	0.327	-0.261	-0.073	-0.425*	-0.646**	-0.470*	-0.507**	0.956**	1	
**SO_4^{2-}**	-0.304	-0.183	0.140	-0.328	0.266	-0.075	-0.210	-0.045	0.029	0.285	-0.166	-0.009	-0.451*	-0.619**	-0.402*	-0.650**	0.763**	0.876**	1

Same acronyms as indicated before.

*: Correlation is significant at the 0.05 level (2-tailed).

**: Correlation is significant at the 0.01 level (2-tailed).
