# Supplementary material for: Spatial heterogeneity of physicochemical properties explains differences in microbial composition in arid soils from Cuatro Cienegas, Mexico
Source: PeerJ. 2016 Sep 8;4:e2459. doi: 10.7717/peerj.2459 (PMC5018672; doi:10.7717/peerj.2459)
Supplement: Table S2 [file peerj-04-2459-s006.pdf]

Table S2. Results of statistical analysis (p-values) for the two primary principal components (PCs) score related to the biogeochemical parameters.

	**PC1**	**PC2**	**r^2**	**Pr (>r)**
TC	-0.98694	-0.1611	0.1233	0.205794
TN	-0.48554	0.87421	0.6359	0.000999***
C:N	0.35451	-0.93505	0.4719	0.001998**
TP	-0.82747	-0.56151	0.5066	0.000999***
NH_4	0.46797	-0.88374	0.4752	0.000999***
NO_3	0.00021	-1	0.391	0.000999***
DOC	-0.74922	-0.66232	0.2479	0.031968*
DON	-0.31693	0.94845	0.5128	0.000999***
DOC:NOD	0.11516	-0.99335	0.3917	0.003996**
DOP	0.7989	0.60147	0.232	0.033966*
pH	-0.96627	-0.25752	0.327	0.00999**
CE	-0.66237	-0.74917	0.0732	0.40959
Mg^{2+}	-0.88973	-0.45649	0.5688	0.000999***
Ca^{2+}	-0.97013	0.24259	0.5736	0.000999***
Na^{+}	-0.87376	-0.48636	0.5452	0.000999***
K^{2+}	-0.89145	-0.45312	0.6021	0.000999***
HCO_3^-	0.94794	-0.31845	0.7406	0.000999***
Cl^-	0.98365	-0.18008	0.7713	0.000999***
SO_4^{2-}	0.99998	0.00602	0.671	0.000999***
Exp. Var.	54.29%	34.32%		

Exp. Var.: Explained variance.

Significance codes: ***, 0.001; **, 0.01; *, 0.05.

P values based on 1000 permutations.
