# Supplementary material for: Spatial heterogeneity of physicochemical properties explains differences in microbial composition in arid soils from Cuatro Cienegas, Mexico
Source: PeerJ. 2016 Sep 8;4:e2459. doi: 10.7717/peerj.2459 (PMC5018672; doi:10.7717/peerj.2459)
Supplement: Table S3 [file peerj-04-2459-s007.pdf]

Table S3. Results of statistical analysis (p-values) of the detrended correspondence analysis (DCA) for the two ordination scores related to the biogeochemical parameters.

	**DCA1**	**DCA2**	**r^2**	**Pr (>r)**
TC	-0.37529	0.92691	0.1138	0.25974
TN	-0.90475	0.42594	0.2365	0.042957*
C:N	0.99081	0.13525	0.1449	0.137862
TP	-0.7869	-0.61708	0.0547	0.480519
NH_4	0.90437	-0.42674	0.1038	0.288711
NO_3	0.88946	-0.45702	0.1302	0.183816
DOC	-0.81726	0.57626	0.1014	0.301698
DON	-0.98655	0.16345	0.2349	0.057942^{+}
DOC:NOD	0.95595	0.29352	0.0763	0.400599
DOP	0.98925	0.14625	0.0165	0.846154
pH	-1	-0.00071	0.072	0.414585
CE	-0.04894	-0.9988	0.0047	0.955045
Mg^{2+}	-0.8232	-0.56775	0.0796	0.35964
Ca^{2+}	-0.99461	-0.10368	0.3667	0.006993**
Na^{+}	-0.54688	-0.83721	0.0805	0.381618
K^{2+}	-0.89132	-0.45337	0.3119	0.014985*
HCO_3^{-}	0.88152	0.47215	0.5533	0.000999***
Cl^{-}	0.87362	0.48661	0.4973	0.000999***
SO_4^{2-}	0.87661	0.4812	0.6862	0.000999***

Significance codes: ***, 0.001; **, 0.01; *, 0.05; $^{+}$, 0.1.

P values based on 1000 permutations.
