# Supplementary material for: Spatial heterogeneity of physicochemical properties explains differences in microbial composition in arid soils from Cuatro Cienegas, Mexico
Source: PeerJ. 2016 Sep 8;4:e2459. doi: 10.7717/peerj.2459 (PMC5018672; doi:10.7717/peerj.2459)
Supplement: Table S4 — Shows the distribution of OTUs diversity in relation to the soil abiotic variables and the respective fit values (R-squared, p-value, vif and AIC). [file peerj-04-2459-s008.pdf]

Table S4. Multiple linear regression model of the distribution of OTUs abundance in relation to the soil abiotic variables and the respective fit values (R-squared, p-value, vif and AIC).

Call:
lm(formula = log(OTUs) ~ log(DON) + Ca + K + log(SO4))

Residuals:

Min	1Q	Median	3Q	Max
-0.45504	-0.17456	0.07136	0.15599	0.38821

Coefficients:

	Estimate	Std. Error	t value	Pr(>|t|)
(Intercept)	7.5194	1.0555	7.124	2.41e-06 ***
log(DON)	0.5895	0.1983	2.972	0.00899 **
Ca	-4.9182	1.2889	-3.816	0.00152 **
K	-0.7555	0.2995	-2.523	0.02261 *
log(SO4)	-0.6553	0.2022	-3.241	0.00511 **

Signif. codes: 0 ‘***’ 0.001 ‘**’ 0.01 ‘*’ 0.05 ‘.’ 0.1 ‘ ’ 1

Residual standard error: 0.2763 on 16 degrees of freedom
Multiple R-squared: 0.5875, Adjusted R-squared: 0.4719
F-statistic: 5.467 on 4 and 16 DF, p-value: 0.005706

Shapiro-Wilk normality test:
data: resid(model)
W = 0.94164, p-value = 0.2348

Multicolinearity diagnostic using Variance Inflation Factor (VIF):

	log(DON)	Ca	K	log(SO4)
VIF	1.929899	2.160122	1.439595	1.741604

Akaike’s information criterion (AIC): 11.869
